# The Risk of Amenorrhea Is Related to Chemotherapy-Induced Leucopenia in Breast Cancer Patients Receiving Epirubicin and Taxane Based Chemotherapy

**DOI:** 10.1371/journal.pone.0037249

**Published:** 2012-05-16

**Authors:** Wenbin Zhou, Qiang Ding, Xiuqing Liang, Zhongyuan He, Xiaoming Zha, Xiaoan Liu, Shui Wang

**Affiliations:** Department of Breast Surgery, The First Affiliated Hospital with Nanjing Medical University, Nanjing, Jiangsu, China; Sudbury Regional Hospital, Canada

## Abstract

**Background:**

Chemotherapy-induced amenorrhea (CIA) is common in young breast cancer patients. The incidence of CIA associated with regimens involving epirubicin and taxane was not well known. Furthermore, previous studies suggested leucopenia and amenorrhea may reflect inter-individual variations in pharmacokinetics. The purpose of this study was to investigate the association between leucopenia after first cycle of chemotherapy and CIA in young breast cancer patients receiving epirubicin and taxane based chemotherapy. Furthermore, the incidence of CIA was also assessed.

**Methodology and Principal Findings:**

Between October 2008 and March 2010, 186 consecutive premenopausal patients, treated with epirubicin and taxane based chemotherapy, were recruited. Information about CIA was collected by telephone and out-patient clinic. Of these 186 patients, data from 165 patients were included and analyzed. Of all 165 patients, CIA occurred in 72 patients (43.64%). In multivariate analysis, age older than 40 y (OR: 16.10, 95% CI: 6.34–40.88, *P*<0.001) and previous childbearing (OR: 3.17, 95% CI: 1.06–9.47, *P* = 0.038) were significantly associated with probability of CIA. Compared to patients treated without taxane, patients treated with taxane-contained regimens did not have a significantly higher rate of CIA (*P*>0.05). The rate of CIA in leucopenia group (52.56%) was significantly higher than that in normal leukocyte group (34.62%) (*P* = 0.024). In patients treated with a FEC regimen (cyclophosphamide, epirubicin and 5-fluorouracil), the rate of CIA in leucopenia group (59.57%) was significantly higher than that in normal leukocyte group (36.84%) (*P* = 0.037).

**Conclusions:**

Age at diagnosis and previous childbearing were both found to significantly increase the risk of CIA, whereas additional taxane was not associated with increased rate of CIA. Importantly, leucopenia after first cycle of chemotherapy was associated with increased risk of CIA, which suggested that leucopenia may be an early predictor of chemotherapy-induced infertility.

## Introduction

Breast cancer is a worldwide malignant disease. Adjuvant chemotherapy can significantly improve disease-free survival (DFS) and overall survival (OS) for early breast cancer patients [1]. However, adjuvant chemotherapy can cause many long-term side effects, such as chemotherapy-induced amenorrhea (CIA) [2]–[4]. CIA is associated with menopause symptoms, infertility, and prolonged exposure to menopausal risks such as osteoporosis [5]. More and more young patients are concerned about preserving their fertility. Therefore, it is important to identify individuals who are with high risk of amenorrhea after chemotherapy.

Many factors, including patients' age, dosage of chemotherapy, and schedule of chemotherapy are associated with the risk of CIA. Usually, old patients had a high risk of CIA due to a reduced number of active ovarian follicles present with increasing age [6]. Chemotherapy regimens used for the treatment of breast cancer include cyclophosphamide, epirubicin, fluorouracil, docetaxel, and paclitaxel. Cyclophosphamide has repeatedly been demonstrated to be quite toxic to the ovaries [7], [8]. Most regimens contain more than one drug. The incidence of CIA associated with regimens involving cyclophosphamide or anthracyclines ranges from 53–89% [9]. Previous studies showed discordant results in the incidences of taxane-induced amenorrhea. Some studies showed that adding taxane to doxorubicin increased the risk of amenorrhea [10]–[13]. However, adding taxane to epirubicin did not increase the risk of amenorrhea in other studies [14], [15]. Epirubicin was widely used in adjuvant chemotherapy for early breast cancer patients [16]–[18]. The CIA rate with epirubicin and taxane based chemotherapy is not well known.

When the above factors were adjusted, CIA rates may be still different in different individuals. Inter-individual variations in pharmacokinetics, influence the degree of the toxicity, may be inner factors. Leucopenia, chemotherapy-induced bone marrow toxicity, is common after chemotherapy, and it may be positively related to the prognosis [19]–[23]. Furthermore, CIA was associated with improved survival [24]. These studies suggested that both leucopenia and amenorrhea may reflect inter-individual variations in pharmacokinetics and may be markers of high bio-availability. Rosendahl and colleagues [23] reported lower leukocyte nadir in response to FEC regimen (cyclophosphamide, epirubicin and 5-fluorouracil) were associated with increased risk of amenorrhea in younger patients. Leucopenia may be an early predictor for CIA. However, the association between leucopenia after first cycle of chemotherapy and taxane-contained regimens induced amenorrhea is far less known.

In this study, we aimed to investigate the association between leucopenia after first cycle of chemotherapy and CIA in young breast cancer patients receiving epirubicin and taxane based chemotherapy. A secondary aim was to evaluate the impact of epirubicin and taxane based regimens on the rate of CIA in Chinese patients. Furthermore, other potential risk factors of CIA were also assessed.

## Materials and Methods

### Patients

The study was conducted according to ethical considerations for observational retrospective studies, and this study was in compliance with the Helsinki Declaration. All breast cancer patients provided written informed consent for their clinical data to be reviewed by us. Between October 2008 and March 2010, 186 consecutive premenopausal patients, treated with epirubicin and taxane based chemotherapy, were recruited at our hospital. Patient data were included in this retrospective study when they met the following criteria: (1) not receiving bilateral oophorectomy or luteinizing hormone releasing hormone (LHRH) agonists; (2) not receiving chemotherapy previously and (3) without recurrent disease in 12 months. Information about CIA was collected by telephone and out-patient clinic. The following information was collected: (1) after which cycle of chemotherapy did the patients experience amenorrhea; (2) when did the menstruation recover; (3) how many times did the menstruation occur after amenorrhea. Since 5 of the 186 patients had recurrent disease within 12 months, 11 were lost during follow-up, 5 had been treated with LHRH, data from 165 patients were included and analyzed at last. The median follow-up time from the initiation of chemotherapy was 26 months (range, 18–35 months).

The chemotherapy regimens were determined based on National Comprehensive Cancer Network (NCCN) guidelines and included: (1) FEC (5-fluorouracil 500 mg/m^2^ on day 1, epirubicin 75 mg/m^2^ on day 1, and cyclophosphamide 500 mg/m^2^ on day 1) every 3 weeks for six cycles; (2) sequential-ECT: CE every 3 weeks for four cycles followed by T (docetaxel 75 mg/m^2^ on day 1 every 3 weeks or paclitaxel 175 mg/m^2^ on day 1 every 2 weeks) for four cycles; (3) FEC-T: FEC every 3 weeks for three cycles followed by docetaxel every 3 weeks for three cycles; and (4) concurrent-ECT (docetaxel 75 mg/m^2^) on day 1 every 3 weeks for six cycles. To reach 100% dose, patients treated with taxane were granulocyte colony stimulating factor (G-CSF) supported. All the patients were G-CSF supported in the case of leucopenia. White blood cell (WBC) count and neutrophilic granulocyte count were assessed on day 7 after chemotherapy. Tamoxifen was given as adjuvant endocrine therapy after chemotherapy when patients were positive for estrogen receptor (ER) and/or progesterone receptor (PR). Radiotherapy was administered to some patients according to NCCN guidelines. Other clinical information was collected for this study, including age, tumor size, nodal involvement, hormone receptor status, and pathology.

### Definitions of leucopenia and CIA

Premenopausal status was defined according to NCCN guidelines. The patients with recovery menstruation were defined as the patients in whom regular menstruation occurred more than three times after temporary amenorrhea [25]. CIA was defined as the cessation of menses for at least 12 months after the end of chemotherapy [23], [26], [15]. Temporary amenorrhea was defined as the menses recovered in 12 months after the end of chemotherapy. Leucopenia in this study was defined as WBC less than 3.0×10^9^/L and/or neutrophilic granulocyte count less than 1.5×10^9^/L on day 7 after first cycle of chemotherapy. Otherwise, normal leukocyte was defined as WBC more than 3.0×10^9^/L and neutrophilic granulocyte count more than 1.5×10^9^/L on day 7 after first cycle of chemotherapy.

### Statistical analysis

In this study, percentiles, median, and range were analyzed for each continuous variable. Differences between subgroups were examined using the chi-square test. The candidate explanatory variables in the multivariate analysis of CIA onset were: age at diagnosis, chemotherapy regimen, childbearing, and use of tamoxifen. Logistic regression was used for multivariate analysis. *P*<0.05 was considered significant. All analyses were performed using the software STATA version 11.0 (Computer Resource Center, America).

## Results

In all, 165 patients were included in this study. Of these patients, 85 were treated with a FEC regimen, 52 with a sequential-ECT regimen, 19 with a FEC-T regimen, and 9 with a concurrent-ECT regimen. The patients' characteristics are shown in Table 1. The median age of these patients was 42 y (range, 26–53 y). Hormone receptors were positive for 106 patients, to whom tamoxifen was given. Of these 165 patients, 136 patients were diagnosed with invasive ductal carcinomas. Taxane-contained regimens were administrated to most patients with lymph node involved.

**Table 1:** Patients' characteristics.

Characteristic	N (%)
Age≤40 y	
Yes	64 (38.79%)
No	101 (61.21%)
Tumor size	
T1	84 (50.91%)
T2	66 (40.0%)
T3	5 (3.03%)
NA	10 (6.06%)
Nodal status	
Positive	67 (40.61%)
Negative	87 (52.73%)
NA	11 (6.67%)
Hormone receptor status	
Positive	106 (64.24%)
Negative	59 (35.76%)
Pathology	
IDC	136 (82.42%)
Other	29 (17.58%)

NA, not available; IDC, invasive ductal carcinoma.

### Risk factors of CIA

Almost all patients had amenorrhea, most of which came up after first three cycles of chemotherapy. Of all 165 patients, CIA occurred in 72 patients (43.64%). Most patients with temporary amenorrhea experienced resumption of menstruation in about 8 months after the end of chemotherapy.

The incidences of CIA according to different variables were shown in Table 2. The rate of CIA was 64.36% (65/101) in patients older than 40 y, while the rate was only 10.94% (7/64) in patients 40 y and younger. For 85 patients treated with a FEC regimen, CIA occurred in 42 patients (49.41%); the CIA rates in patients treated with a sequential-ECT, FEC-T and concurrent-ECT were 42.31% (22/52), 21.05% (4/19) and 44.44% (4/9), respectively. The impact of previous childbearing on the incidence of CIA was also analyzed. Rates of CIA were 28.57%, 40.88% and 63.16% for patients with no child, one child and more than one child, respectively. In addition, the incidences of CIA in patients treated with tamoxifen and without tamoxifen were 44.34% and 42.37%.

**Table 2:** The incidence of CIA in different groups.

Variable	No. CIA (%)
Age at diagnosis, y	
>40 y	65/101 (64.36%)
≤40 y	7/64 (10.94%)
Chemotherapy regimen	
FEC	42/85 (49.41%)
sequential-ECT	22/52 (42.31%)
FEC-T	4/19 (21.05%)
concurrent-ECT	4/9 (44.44%)
Previous childbearing*	
0	2/7 (28.57%)
1	56/137 (40.88%)
≥2	12/19 (63.16%)
Tamoxifen use	
Yes	47/106 (44.34%)
No	25/59 (42.37%)

* the information about childbearing of two patients was not available.

In multivariate analysis (Table 3), age older than 40 y (odds ratio (OR): 16.10, 95% confidence interval (CI): 6.34–40.88, *P*<0.001) and previous childbearing (OR: 3.17, 95% CI: 1.06–9.47, *P* = 0.038) were significantly associated with probability of CIA. Patients treated with tamoxifen had a trend towards higher rate of CIA (OR: 1.21, 95% CI: 0.54–2.74, *P* = 0.647) compared with patients not receiving tamoxifen. Compared to patients treated with a FEC regimen, patients treated with taxane-contained regimens did not have a significantly higher rate of CIA (*P*>0.05 for all three regimens).

**Table 3:** Multivariate analysis of CIA.

Variable	OR	95% CI	*P*-value of CIA
Age(>40 y vs. ≤40 y)	16.10	6.34–40.88	**<0.001**
Chemotherapy regimen			
FEC	1.0	Reference	
sequential-ECT	0.30	0.08–1.14	0.077
FEC-T	0.83	0.36–1.93	0.670
concurrent-ECT	1.29	0.23–7.20	0.770
Childbearing(≥2 vs.1 vs.0)	3.17	1.06–9.47	**0.038**
Tamoxifen use(yes vs. no)	1.21	0.54–2.74	0.647

OR, odds ratio; CI, confidence interval.

### The relationship between leukopenia and CIA

The patients treated with concurrent-ECT regimen were G-CSF supported after first cycle of chemotherapy, so these patients were excluded from the analysis of the relationship between leucopenia and CIA. In all, 156 patients were included for this analysis (Table 4).

**Table 4:** Patient characteristics in leucopenia group and normal leukocyte group.

Variable	Normal leukocyte	Leucopenia	*P* value
CIA			
Yes	27	41	**0.024**
No	51	37	
Age			
≤40 y	36	24	0.055*
>40 y	42	54	
Childbearing			
0	4	2	0.935*
1	62	67	
≥2	11	8	
NA	1	1	
Chemotherapy regimen			
FEC	38	47	Reference
sequential-ECT	30	22	0.155*
FEC-T	10	9	0.615*

NA, not available;

* *P*>0.05 for multivariate analysis of leucopenia.

Of these 156 patients, 78 (50%) experienced leucopenia after first cycle of chemotherapy. In multivariate analysis, age at diagnosis, chemotherapy regimen, and previous childbearing were not associated with leucopenia after first cycle of chemotherapy (*P*>0.05). The rate of CIA in leucopenia group (52.56%) was significantly higher than that in normal leukocyte group (34.62%) (F = 5.11, *P* = 0.024). In patients treated with a FEC regimen, the rate of CIA in leucopenia group (59.57%) was significantly higher than that in normal leukocyte group (36.84%) (F = 4.34, *P* = 0.037). Patients treated with a sequential ECT regimen in leucopenia group (50%) had a trend towards higher CIA rate compared with patients in normal leukocyte group (36.67%), but no significant difference was observed (*P*>0.05) due to small sample size.

Because there was a trend toward more leucopenia in older patients (*P* = 0.055), the association between leucopenia and CIA was also analyzed in patients older than 40 y or 40 y and younger treated with a FEC regimen. In patients older than 40 y treated with a FEC regimen, the rate of CIA in leucopenia group (25/32) was significantly higher than that in normal leukocyte group (12/23) (F = 4.09, *P* = 0.043). In patients 40 y and younger treated with a FEC regimen, the rate of CIA in leucopenia group (3/15) was not significantly higher than that in normal leukocyte group (2/15) (*P*>0.05). However, the association between leucopenia and CIA was not analyzed in patients older than 40 y or 40 y and younger treated with other two regimens due to small sample size.

## Discussion

Chemotherapy-induced premature menopause and infertility influence patients' life quality seriously. It is important to investigate the risk factors of CIA. The CIA risk of epirubicin and taxane based chemotherapy is not well known. Our results demonstrated that the risk of CIA was significantly related to age at diagnosis and previous childbearing. Furthermore, compared with the FEC regimen, taxane based regimen did not show a higher rate of CIA. Importantly, leucopenia after first cycle of chemotherapy was associated with CIA in patients treated with epirubicin and taxane based chemotherapy.

The definitions of CIA were different in previous studies. CIA was defined as the absence of menses for at least three consecutive months from the point of breast cancer diagnosis in some studies [9], [10], while some authors defined it as the cessation of menses for 12 months from the beginning of chemotherapy [27], [5]. The incidence of CIA in the extant literature may be influenced by inconsistent definitions. Most patients receiving chemotherapy will experience irregular menses and many women may experience a return of menses during the first year after chemotherapy [15], [23]. We defined CIA as the cessation of menses for at least 12 months after the end of chemotherapy, which can evaluate the destruction of the ovarian reserve accurately.

Previous studies reported discordant results in the incidences of taxane-induced amenorrhea [5], [11], [10], [14], [15]. The incidence of CIA in patients receiving epirubicin and taxane based regimens is not well known. In this study, epirubicin and taxane based regimens (sequential-ECT, concurrent-ECT, and FEC-T) did not have a higher risk of CIA than the FEC regimens, which was similar to previous report of TE (docetaxel and epirubicin) regimen [15]. Furthermore, additional docetaxel to FEC did not increase the risk of amenorrhea in PACS01 trial [14]. However, compared to adriamycin based chemotherapy, additional taxane increased the risk of amenorrhea in previous studies [12], [11], [10]. The interactions of these drugs may be responsible for the difference. Future clinical trials are needed to confirm this interesting finding.

This study showed that age was still the most important risk factor of CIA, which was consistent with previous studies. In addition, we found that previous childbearing was significant associated with CIA, which was the same as the previous study [28]. The highly increased human chorionic gonadotropin (HCG) in pregnancy, with the similar effects as follicle-stimulating hormone (FSH) [29], can increase preantrla follicles differentiation. The increased growth factors in pregnancy can also induce the maturation of primordial follicles [30]. However, it is known that FSH is down-regulated in pregnancy. Furthermore, high estrogen concentrations in pregnancy significantly increased vessel endothelial area [31]. Although the long-term effects of these hormones to the ovary were not clear, the structure of the ovary in patients with previous childbearing may be different from that in nulliparous patients due to great changes of hormones in pregnancy. The changes in the ovary may contribute to the different CIA rates between patients with previous childbearing and nulliparous patients, but the accurate underlying mechanisms are still not known. Previous study suggested that the drop in Anti Müllerian Hormone (AMH) may play a role in increased recruitment of preantral follicles [32]. Measuring the dynamic changes in AMH before and after chemotherapy in these patients may provide information about the mechanisms underlying different rates of CIA. This finding was obtained from these two retrospective studies with small sample size, so future large randomized clinical trials are needed to confirm this interesting result. However, previous childbearing should be considered as an important risk factor of CIA when chemotherapy was given. Patients treated with tamoxifen had a trend towards higher incidence of CIA, but no significant difference was observed in this study. AMH can be used to evaluate the pool of resting primordial follicles in the ovaries. A previous study suggested that the levels of AMH in patients treated with, or without, tamoxifen were not different [23], so tamoxifen may increase the risk of amenorrhea but not ovarian failure. Randomized controlled trials with large sample size should be performed to determine the risk factors of CIA.

Previous studies demonstrated that chemotherapy-induced amenorrhea and leucopenia were both associated with improved survival [24], [19]–[23]. Therefore, amenorrhea and leucopenia may be markers of high bio-availability. To our knowledge, this is the first report of a relationship between leucopenia after first cycle of chemotherapy and CIA in patients receiving epirubicin and taxane based chemotherapy. Patients with high bio-availability may have increased risk of infertility. More and more young breast cancer patients are concerned with maintaining their fertility [33]. Because leucopenia may be an early predictor of chemotherapy-induced infertility, methods of fertility preservation should be considered as early as possible for patients with leucopenia.

On the other hand, several limitations were present in this study. First, this is a retrospective study with marginally significant differences found with regard to age and leucopenia, future studies would be required to investigate these. Second, only Chinese patients were included in this study. Future studies should be taken in other populations. Third, endocrinological data were not assessed in this study. Since oligo-amenorrhoea may be caused by tamoxifen, multivariate analysis was used to assess the risk factors of CIA in this study.

Our study suggested age at diagnosis and previous childbearing were both found to significantly increase the risk of CIA. Compared to patients treated with the FEC regimen, additional taxane did not significantly increase the rate of CIA. Importantly, leucopenia after first cycle of chemotherapy was associated with increased risk of CIA. When patients want to maintain their fertility, our results should be considered. Methods of fertility preservation should be administrated as early as possible for patients with leucopenia.
